# Staged reduction of neglected transscaphoid perilunate fracture dislocation: A report of 16 cases

**DOI:** 10.1186/1749-799X-7-19

**Published:** 2012-05-20

**Authors:** Bhavuk Garg, Tarun Goyal, Prakash P Kotwal

**Affiliations:** 1 Department of Orthopaedics, All India Institute of Medical Sciences, 110029, New Delhi, India

## Abstract

**Background:**

Transscaphoid perilunate fracture dislocation is a rare injury and can be easily missed at the initial treatment. Once ignored, late reduction is not possible and needs extensive dissection. An alternative treatment such as proximal row carpectomy may be required for neglected injuries, but surgical outcome is not as good as that of an early reduction. We aim to present an alternative technique of staged reduction and fixation in patients of neglected transscaphoid perilunate dislocations and study its outcome.

**Material & Methods:**

16 cases (14 males & 2 females) with neglected transscaphoid perilunate fracture dislocation (> 3 month old) were treated with staged reduction. Mean duration between injury and surgery was 4.5 months. In first stage an external fixator was applied across the wrist and distraction was done at 1 mm/day. Second surgery was done through dorsal approach and we were able to reduce all the fractures & dislocations. Herbert screws and K wires were used for fixation.

**Results:**

The mean duration between two surgeries was 2.4 weeks (range 2–4 weeks). 9 cases had excellent results, 5 had good result. Two patients developed reflex sympathetic dystrophy and had fair results.

**Conclusion:**

Staged reduction should be considered for neglected transscaphoid perilunate dislocations. If properly executed, a good functional pain free range of motion is the usual outcome.

## Background

Transscaphoid perilunate dislocations are rare injuries. It is estimated that upto 25% of these injuries are diagnosed late [1]. This percentage is still higher in developing countries due to lack of awareness and facilities for diagnosis and treatment.

Treatment of neglected injuries carries a poorer prognosis as compared to fresh injuries [2,3]. Late reduction of perilunate dislocations is difficult and after 3 months salvage procedures such as proximal row carpectomy, excision of the lunate or wrist arthrodesis have been recommended [4]. Most of the patients treated with these methods do not achieve good functional outcomes and have limitations of activities due to reduced range of motion, pain, and loss of grip strength. Treatment of these neglected injuries with open reduction and internal fixation would theoretically give better functional results as compared to salvage procedures [1,3,5]. However, this often requires extensive soft tissue releases to achieve reduction, leading to scarring and loss of vascularity of carpal bones, thus compromising the outcomes. We propose that staged reconstruction of these injuries is superior to single stage surgical release. Soft tissues and carpal bones are gradually distracted with a spanning external fixator followed by open reduction and fixation using dorsal approach.

We aim to study functional outcomes in 16 patients treated this novel technique over a mean follow up of 4.2 years. Search of medical literature on this topic did not reveal any series on neglected dislocations using this technique.

## Methods

**Figure 1:**
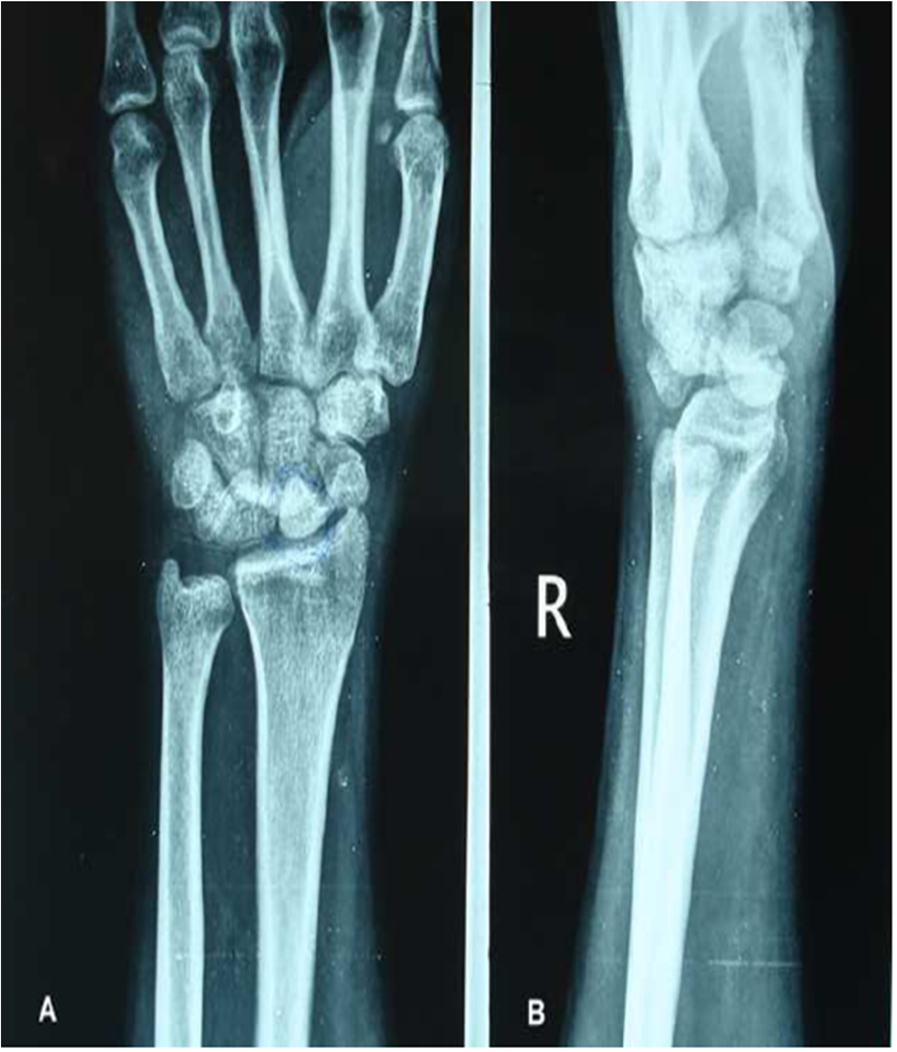
Preoperative radiograph showing a neglected transscaphoid perilunate dislocation; (A) Anteroposterior and (B) Lateral views.

Between January 2004 and July 2007, 16 patients (male: females 14:2; mean age 28 years, range 18–38 years) with neglected perilunate dislocation (more than 3 months old) were treated using this technique. Approval for the study was obtained by the Institute Ethics Committee. Diagnosis of perilunate dislocation was established on standard anteroposterior, lateral and stress radiographs. Figure 1 shows preoperative anteroposterior and lateral radiographs of a neglected transscaphoid preilunate dislocation. Computed tomography scans with 3D reconstructions and fine 1 mm cuts were done in all the patients to look at the size of the fragments and anatomy of the carpal bones. Magnetic resonance imaging was also obtained to look for vascular status of the scaphoid.

### Surgical technique

**Figure 2:**
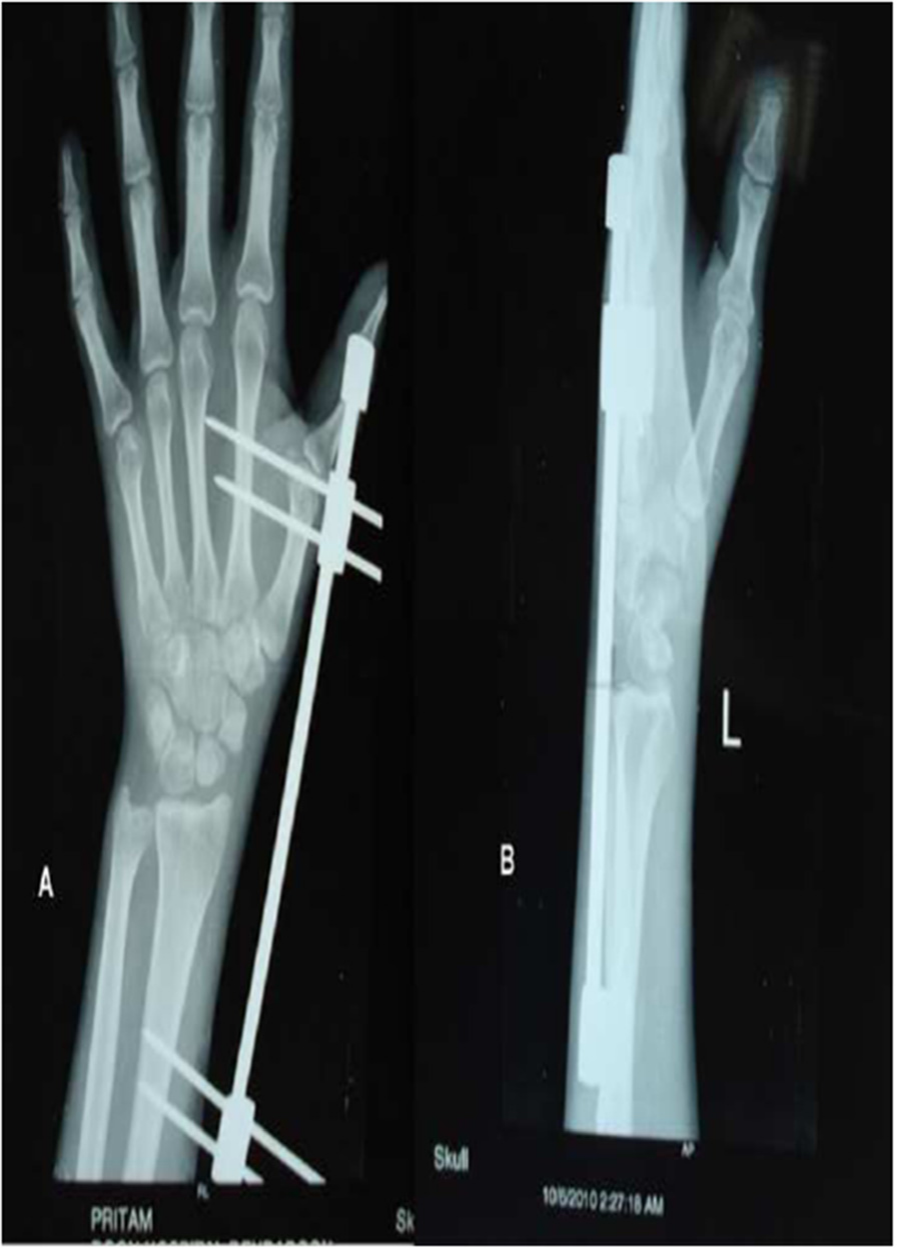
(A & B): First stage of reconstruction with a spanning external fixator. Carpal bones are gradually distracted to restore length and alignment using the fixator.

All these patients were treated in two stages. In the first surgery a spanning external fixator was applied across the carpal bones with two pins in dorsolateral aspect of the radius and two pins in the second metacarpal. This was used for gentle distraction across the carpal bones at the rate of 1 mm per day. Patients were followed up with weekly radiographs following surgery to judge adequate distraction. Distraction was termed adequate when there was no change in the intercarpal alignment on further distraction with the proximal pole of capitate coming to lie at the level of distal part of lunate in the lateral radiograph and radiocarpal joint started distracting (Figure 2).

This was followed by a second stage open reduction and internal fixation of the carpal bones and intercarpal ligament reconstruction using dorsal approach. A dorsal transverse incision was given about 1 cm distal to the radiocarpal joint extending from the radial styloid to the ulnar styloid.

**Figure 3:**
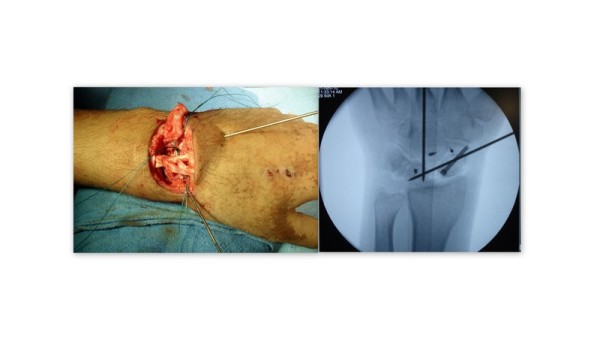
Intraoperative image showing placement of K wires to hold the fracture- dislocation and placement of suture anchors. Placement of headless screw and suture anchors can be seen on intraoperarive radiograph.

Extensor retinaculum was incised parallel to the skin incision. Extensor tendons are retracted medially and laterally to gain exposure of the dorsal joint capsule. Dorsal capsule is opened along the dorsal intercarpal ligament making a radial V shaped based flap. The fracture sites are now reduced and provisionally fixed with k wires. Definitive fixation was achieved with Herbert screws. Repair of the scapholunate and scaphocapitate intercarpal ligament was carried out in all the cases using suture anchors. In 4 cases the ligaments were found to be highly redundant and strength of repair was doubtful. In these cases the ligaments were buttressed with a capsular flap from the dorsal capsule. Vascularity of scaphoid was assessed intraoperatively using a 1.5 mm K wire, which showed bleeding bone in all the cases. All the fractures were bone grafted using iliac crest bone graft. Intraoperative photograph showing placement of suture anchors and K wires is shown in Figure 3.

### Rehabilitation

In the immediate postoperative period limb was rested in a volar below elbow slab for three weeks. Mobilisation of the shoulder, elbow and digital articulations were started in the immediate postoperative period. Active assisted and passive mobilisation of the wrist was started at 2 weeks. Intrinsic muscle strengthening and grip exercises were begun at 2 months. Return to full activities or sports was allowed at 4–6 months.

### Follow up

**Figure 4:**
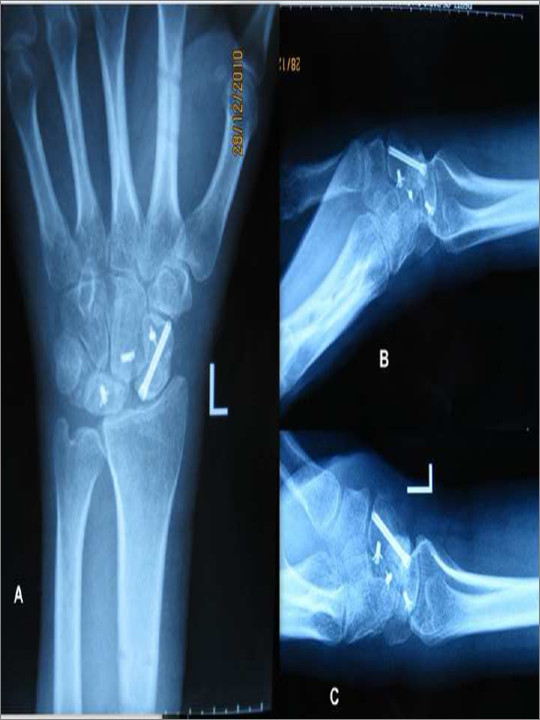
(A)Follow up radiographs showing adequate reduction using Herbert screws and suture anchors. (B & C) Reduction is maintained on radiographs obtained in dorsifexion and plamer flexion.

Patients were followed up at 1 month, two month 4 months, six months and then at 6 monthly interval. Mean duration of follow up was 4.2 years (range 2.5 years to 5.8 years). Clinically patients were evaluated for pain, range of motion of the wrist joint in dorsiflexion and palmer flexion, and radial and ulnar tilt. Grip strength was measured using a dynamometer. Functional assessment was done using Mayo’s scoring system for wrist which is based on pain, functional status, range of motion, and grip strength [6,7]. Pain was also subjectively graded as permanent pain, occasional pain affecting daily activities, pain on strenuous activities and no pain. Plain anteroposterior and lateral radiographs of the wrist joint were obtained to look for any residual malalignment, loss of fixation, evidence of avascular changes or degenerative changes in the radiocarpal and midcarpal articulation (Figure 4). Radiological evaluation was carried out using the system given by Herzberg et al [1]. Preoperative and postoperative scapholunate and radiolunate angles were also measured for adequacy of reduction.

## Results and discussion

Mean duration between injury and surgery was 4.5 months (range 3.5 to 7 months). Mode of injury and presenting complaints are summarised in Table 1. Adequate reduction was seen in all the patients. Mean scapholunate and radiolunate angles were 52 degrees (range 45 to 56 degrees) and 11 degrees (range 8 to 13 degrees) on the immediate postoperative radiographs and 57 degrees (range 45 to 62 degrees) and 18 degrees (10 to 23 degrees) at 3 years follow up. The mean duration between two surgeries was 2.4 weeks (range 2–4 weeks).Union of the scaphoid fractures was seen in all the 16 cases. Subjectively 9 patients reported no pain, 5 patients had pain on strenuous activities and 2 patients had pain on routine activities. Mean Mayo score was 78 (70 to 90). Nine cases had excellent results, 5 had good results and 2 had fair result. Mean grip strength at 3 years follow up was 41 kg (range 34–49 kg). This was 88% of the normal side. Mean wrist flexion was 65 degrees (55 to 70 degrees), extension was 50 degrees (45 to 60 degrees), radial tilt was 20 degrees (15 to 25 degrees) and ulnar tilt was 30 degrees (20 to 45 degrees). The functional outcome of these patients has been summerised in Table 2. Recovery of median nerve function was seen in all the patients at follow up.

**Table 1:** Mechanism of injury and presenting signs and symptoms

**Mode of injury**	**No of patients**
RTA	12
Fall from height	3
Sports injuries	1
**Presenting symptoms and signs**	
Pain	14
Swelling	12
Stiffness	16
Median nerve symptoms	4

**Table 2:** Functional outcome of patients at follow up

**Parameter**	**No of patients**
**Pain**	
No pain	9
Mild occasional	5
Moderate tolerable	2
**Functional status**	
Return to regular employment	
Restricted employment	
**Range of motion (% of normal side)**	
75- 99%	15
50-75%	1
**Grip strength (% of normal side)**	
75- 99%	18
50-75%	0

Mild degenerative changes were seen in the radiocarpal joint in 3 patients and in midcarpal joint in 6 patients. Avascular changes were seen in the scaphoid in 1 patient. According to Herzberg”s radiological classification, 10 patients were type A, 5 were type B1 and 1 was type C1. There were no wound related complications. Two patients developed reflex sympathetic dystrophy and were treated with stellate ganglion block. Functional scores in both these patients was 70.

There are many reports on treatment of acute perilunate dislocations. But treatment of chronic perilunate dislocations is less often discussed. Late presentation of these injuries not only complicates the treatment but also makes results less satisfactory. Proper evaluation of patients with wrist injuries is thus very important to exclude perilunate instability. Failure to get stress views in suspected cases and unfamiliarity of the treating physician with the normal carpal anatomy can lead to perilunate dislocation being missed at the time of injury. These present later with chronic wrist pain, swelling or median nerve symptoms. Interestingly these patients may regain range of motion with subsidence of pain but function of the wrist joint remains poor with poor grip strength and inability to return to previous activities.

Causes of unsatisfactory results following late fixation of neglected radiocarpal dislocations include scarring, avascularity of the carpal bones particularly the scaphoid either due to the injury or secondary to extensive dissection at the time of open reduction, articular cartilage damage and remodelling of the carpal bones. Avascular necrosis of scaphoid may occur in these cases as in other scaphoid fractures but avascular necrosis of lunate is rare. More common is transient ischemia of the lunate, seen as rediodense lunate amidst osteopenic carpal bones in chronic cases. But this has been found to improve after the dislocation has been reduced and fractures fixed [8].

Contractions of soft tissues, ligaments and neurovascular structures especially the median nerve preclude attempts at reduction of these neglected dislocations. Staged treatment of these injuries has several advantages. The soft tissues are stretched gradually to achieve some alignment of the bones before surgery. This avoids the need of extensive dissection and soft tissue stripping at the time of surgery. Secondly the distractor unloads the carpal bones of excessive and nonanatomic forces. This may help in cartilage regeneration and fibrocartilage formation. Four of our patients had median nerve symptoms at the time of presentation. When distraction was applied using external fixator none of them showed any evidence of worsening of median nerve symptoms. This is probably because the carpal bone are aligned as the distraction is applied and pressure over the median nerve is offloaded.

No clear association is seen between the development of degenerative changes in the radiocarpal and midcarpal articulations and pain. Both our patients who had pain on routine activities had degenerative changes in the midcarpal joints. Out of 5 patients who had pain on sternous activities only 2 patients had degenerative changes in rafiocarpal and midcarpal joints on follow up.

Following open reduction and internal fixation of acute perilunate injuries excellent to good results have been reported in 65 to 80% of the patients [3-5,9]. Mean grip strength as the percentage of the normal side has been reported to be about 80 percent. Mean grip strength in our patients was 88% of the normal side. Mean Mayo’s score was 78. Results of salvage procedures for treatment of these injuries are inferior to internal fixation. Results in literature of open reduction in acute cases, chronic dislocations and salvage procedures for chronic dislocations are summarised in Table 3. Thus staged reduction should be considered for neglected transscaphoid perilunate dislocations.

**Table 3:** Comparison of functional outcomes of patients treated with internal fixation for acute fracture dislocations, for neglected injuries and salvage procedure for neglected injuries

**Study**	**Functional Score**	**Grip strength (percentage of normal wrist)**	**Comments**
**Acute injuries treated with internal fixation**			
Trumble [10]	-	77	
Knoll [11]	-	80	
Souer [12]	71-66	76-67	
Sotereanos [13]	65.5	77	
Hildebrand [14]	66	73	
Herzberg [1]	79	79	
**Chronic injuries treated with salvage procedure**			
Inoue [5]	62.5%- fair	63	Proximal row carpectomy
	37.5%-poor		
Rettig [15]	-	34 (kg)	Proximal row carpectomy
**Chronic injuries treated with internal fixation**			
Siegert et al [3]	6/16 (37.5%) satisfactory results	-	Mean interval between injury and surgery- 17 weeks
Inoue [5]	3 good, 1 fair and 2 poor		Mean interval between injury and surgery- 16 weeks
Kailu L et al [9]	four good, one fair, and one poor	-	Mean interval between injury and surgery- 17 weeks
Komurcu M et al [4]	72.5	26.33 (kg)	Mean interval between injury and surgery- 26 days
**Our study**	75	88	

If properly executed, a good functional pain free range of motion is the usual outcome.

## Conclusion

Neglected transscaphoid perilunate dislocation sre rare but challenging injuries. It is difficult to achieve good results in these injuries. Our experience with two staged reduction and fixation of these injuries is encouraging. This limits the need for soft tissue dissection at the time of surgery and achieves better outcomes as compared to salvage procedures or single stage open reduction.

## Competing interests

The authors declare that they have no competing interests.

## Authors’ contributions

BG, PK and TG were involved in designing the study and acquisition of data. TG and BG contributed to preparation of the manuscript, revising it and giving it the final form. All authors read and approved the final manuscript.
